# Effect of a tailored upper extremity strength training intervention combined with direct current stimulation in chronic stroke survivors: A Randomized Controlled Trial

**DOI:** 10.3389/fresc.2022.978257

**Published:** 2022-08-03

**Authors:** Stephania Palimeris, Yekta Ansari, Anthony Remaud, François Tremblay, Hélène Corriveau, Marie Hélène Boudrias, Marie Hélène Milot

**Affiliations:** ^1^Faculty of Medicine and Health Sciences, School of Physical and Occupational Therapy, McGill University, Montréal, QC, Canada; ^2^BRAIN Lab, Jewish Rehabilitation Hospital, Laval, QC, Canada; ^3^Montreal Center for Interdisciplinary Research in Rehabilitation (CRIR) and CISSS-Laval, Montréal, QC, Canada; ^4^Bruyère Research Institute, Ottawa, ON, Canada; ^5^Faculty of Health Sciences, School of Rehabilitation Sciences, University of Ottawa, Ottawa, ON, Canada; ^6^Faculté de médecine et des sciences de la santé, Université de Sherbrooke, École de réadaptation, Sherbrooke, QC, Canada; ^7^Centre de recherche sur le vieillissement, CIUSSS de l'Estrie-CHUS, Sherbrooke, QC, Canada

**Keywords:** stroke, strengthening exercises, MEP, tDCS, arm impairment, arm function

## Abstract

Strengthening exercises are recommended for managing persisting upper limb (UL) weakness following a stroke. Yet, strengthening exercises often lead to variable gains because of their generic nature. For this randomized controlled trial (RCT), we aimed to determine whether tailoring strengthening exercises using a biomarker of corticospinal integrity, as reflected in the amplitude of motor evoked potentials (MEPs) elicited by transcranial magnetic stimulation (TMS), could optimize training effects in the affected UL. A secondary aim was to determine whether applying anodal transcranial direct current stimulation (tDCS) could enhance exercise-induced training effects. For this multisite RCT, 90 adults at the chronic stage after stroke (>6 months) were recruited. Before training, participants underwent TMS to detect the presence of MEPs in the affected hand. The MEP amplitude was used to stratify participants into three training groups: (1) low-intensity, MEP <50 μV, (2) moderate-intensity, 50 μV < MEP < 120 μV, and (3) high-intensity, MEP>120 μV. Each group trained at a specific intensity based on the one-repetition maximum (1 RM): low-intensity, 35–50% 1RM; moderate-intensity, 50–65% 1RM; high-intensity, 70–85% 1RM. The strength training targeted the affected UL and was delivered 3X/week for four consecutive weeks. In each training group, participants were randomly assigned to receive either real or sham anodal tDCS (2 mA, 20 min) over the primary motor area of the affected hemisphere. Pre-/post-intervention, participants underwent a clinical evaluation of their UL to evaluate motor impairments (Fugl-Meyer Assessment), manual dexterity (Box and Blocks test) and grip strength. Post-intervention, all groups exhibited similar gains in terms of reduced impairments, improved dexterity, and grip strength, which was confirmed by multivariate and univariate analyses. However, no effect of interaction was found for tDCS or training group, indicating that tDCS had no significant impact on outcomes post-intervention. Collectively, these results indicate that adjusting training intensity based on the size of MEPs in the affected extremity provides a useful approach to optimize responses to strengthening exercises in chronic stroke survivors. Also, the lack of add-on effects of tDCS applied to the lesioned hemisphere on exercise-induced improvements in the affected UL raises questions about the relevance of combining such interventions in stroke.

**Clinical trial registry number:**

NCT02915185. https://www.clinicaltrials.gov/ct2/show/NCT02915185.

## Introduction

Worldwide, stroke is the second-leading cause of mortality, with an estimated 6.6 million deaths annually (1) and over 100 million individuals live with residual impairments. One of the most common sequelae following a stroke is hemiparesis of the side contralateral to the affected hemisphere (2). Paresis or muscle weakness can interfere with activities of daily living (ADL), decrease the quality of life (3, 4), and impact interpersonal relationships (5). Residual muscle weakness in the upper limb (UL) is particularly prevalent in stroke survivors affecting more than three-quarters of them (6), with more than half reporting being unable to perform basic ADL, even after intensive rehabilitation therapy (7). Accordingly, improving UL function is a top priority for survivors at the chronic stage of a stroke (8).

European and American Stroke Best Practices recommend strengthening exercises to address residual UL weakness after a stroke (9, 10). Strengthening exercises are considered a key element of rehabilitation interventions for post-stroke paresis by improving muscle strength and motor function (11, 12), and contributing to enhanced motor cortex excitability (13, 14). These effects translate into significant gains in daily use of the affected limb (12). Moreover, recent findings show that patients can still experience significant improvements in arm function in response to training interventions even when they reach the chronic stage after a stroke (≥6 months) (15–18). Yet, individual responses to training are often variables, some showing significant gains while others show either minimal or no response (2, 11). This variability, to a large extent, reflects the fact that exercises are generally prescribed as a “generic” intervention without consideration for individual differences in terms of susceptibility to respond to training and potential for recovery. In clinical settings, therapists will typically build an exercise intervention based on their clients' clinical profiles. However, clients with comparable clinical profiles may exhibit very different potential for recovery (19, 20) and, yet they will often receive the same strength training program.

The corticospinal pathway can be reliably and safely assessed by transcranial magnetic stimulation (TMS) in stroke survivors. The presence of motor evoked potentials (MEPs) in the affected extremity attests to the integrity of the descending projections. Indeed, the presence or absence of MEPs in the affected limbs is a sensitive biomarker to estimate the potential for recovery after a stroke (19). In their PREP algorithm, Stinear et al. (19) demonstrated the validity of using MEPs elicited by TMS in paretic muscles to predict the potential for recovery 72 h post-stroke and to establish realistic goals for rehabilitation for the affected UL. Along the same line, baseline MEP amplitude has been shown to predict individual responses to exercises in patients at the chronic stage of a stroke (21). Thus, there is compelling evidence that TMS responses in the affected hemisphere can provide an index of corticospinal integrity both in the early and later stages after a stroke and that such an index can inform the potential of a given individual to experience good or poor recovery. This raises the interesting question of whether MEPs in the affected extremities could be used to tailor exercise prescription by taking into account the individual's potential for recovery and susceptibility to respond to training. Such an approach could assist therapists in designing more optimal exercise interventions post-stroke.

Non-invasive brain stimulation strategies have also been considered as another means to enhance neuroplasticity and responses to exercise interventions post-stroke. In particular, transcranial direct current stimulation (tDCS) has received much attention recently in the context of post-stroke rehabilitation. By applying a weak current (1–2 mA) through electrodes placed on the scalp, tDCS can increase or decrease neuronal excitability depending on the montage (22, 23). Anodal tDCS tends to increase neural excitability by depolarizing the neurons' membranes, while cathodal tDCS tend to decrease neuronal excitability by hyperpolarizing the neurons' membrane (22–24). Recent studies suggest that tDCS may be more effective in individuals with moderate to mild motor impairment post-stroke (25, 26). In such cases, repeated application of anodal tDCS with various post-stroke rehabilitation interventions translated into significant gains in UL function as well as increased corticospinal excitability (25–34) when compared to sham. Despite these encouraging results, the benefits of combining tDCS with exercise interventions to enhance training-induced effects post-stroke remain unclear.

## Objectives

The primary objective of this study was to determine whether a tailored strengthening intervention could reduce impairments and improve UL function in chronic stroke survivors when participants are regrouped into different intensity-adjusted training levels according to baseline MEP amplitude in the affected arm. The secondary objective was to determine whether combining anodal tDCS of the affected hemisphere with tailored arm strengthening exercises could translate into further benefits in terms of reduced impairments and improved UL function.

Considering that individual responses to training are known to be variable in stroke survivors (20), we anticipated that adjusting the level of strength training intensity between groups according to MEP amplitude would benefit all participants with clinically significant gains in the affected UL function and performance. Also, given the ability of anodal tDCS to enhance motor excitability and promote neuroplasticity post-stroke (26), we anticipated that participants receiving real tDCS would display greater gains in response to training than those receiving a sham.

## Materials and methods

A detailed description of thestudy protocol has been published elsewhere (35). In brief, to be included in this randomized controlled trial, individuals had to meet the following entry criteria: (1) aged ≥18 years; (2) having experienced a single unilateral stroke more than 6 months ago; and (3) having finalized their rehabilitation treatment. Individuals were excluded if presenting: (1) significant spasticity at the affected upper limb (score >3 on the modified Ashworth scale) (36); (2) significant pain intensity at the affected upper limb (≥4/10 on the Visual Analog Pain Scale) (37); (3) major sensory deficit (a score ≤25/34 on the Nottingham Sensory Assessment) (38); (4) presence of hemineglect (>70% of unshaded lines on the same side as the motor deficit on the Line Cancellation Test) (39); (5) apraxia (score >2.5 on the Alexander Test) (40); (6) the presence of a neurological disorder other than a stroke; (7) concomitant orthopedic problems at the affected upper limb; and (8) any contraindication to TMS and/or tDCS.

Prior to training, all participants underwent a clinical evaluation of their affected UL as well as a TMS evaluation. The clinical evaluation, performed by a blinded assessor, included the following primary outcomes: (1) the Fugl-Meyer stroke assessment for the UL impairment (FMA max score = 66) (41), (2) the Box and Block test (BBT; number of blocks in 60 s) (42) to assess dexterity, and (3) Grip strength (average of three trials in kg). The evaluation also included secondary outcomes, which consisted of self-reported quality and quantity of arm use in daily activities (Motor Activity Log /5) (43) and active range of motion (AROM) as measured by standard goniometry for flexion at the affected shoulder and elbow and in extension for the wrist. The TMS evaluation, performed by another blinded trained evaluator, consisted of determining the resting motor threshold (RMT) and MEP amplitude in the affected hemisphere. At all sites, the TMS was delivered using a Magstim stimulator connected to a figure-of-eight coil (Magstim 200^2^/BiStim, Magstim Company, Dyfed, UK). MEPs were recorded using standard procedures to record surface electromyographic activity. For first dorsal interosseous (FDI) recordings, electrodes were placed in a belly-tendon montage (Ottawa site: DE-2.1, Delsys Inc., Boston, MA, USA, Montreal: Neuroline 700, Ambu, Glen Burnie, USA, Sherbrooke: PiCO EMG Cometa, Bareggio, Italy). MEPs were also monitored in the Extensor Carpi Radialis (ECR) with the electrodes placed following anatomical landmarks over the muscle belly. All raw EMG signals were band-pass filtered (6–450 Hz), amplified (× 1,000) and sampled at 2,000 Hz. After amplification and filtering, EMG signals were further relayed to a PC at each site running either custom (Ottawa) or commercially available software (Sherbrooke, Montreal: Spike2 version 8.0, Cambridge Electronic Design Ltd., 2018) for off-line analysis. The TMS evaluation proceeded by first localizing the hotspot for the FDI. Given the difficulty in eliciting MEPs in the affected hemisphere, the hotspot for the FDI was first determined on the unaffected side in all participants. Once determined, the corresponding location on the affected hemisphere was stimulated at a relatively high intensity (60–80% of stimulator output) to elicit responses. When MEPs could be recorded in the FDI, the RMT was then determined using the Motor Threshold Assessment Tool software (MTAT version 2.0) (44). The threshold peak-to-peak amplitude to detect the presence of MEPs in the FDI was set at 50 μV. Next, resting peak-to-peak MEP amplitudes of the FDI were elicited at 130% of RMT and averaged over 10 trials. If no detectable peak-to-peak MEPs amplitude could be elicited in the affected FDI even when stimulating at 100% maximum output of the stimulator, the procedure was repeated using the ECR as the target muscle. If no peak-to-peak MEP amplitudes were detected for the ECR, the participants were considered as having no MEPs. Note that other TMS measures were also performed but these are not reported here and will be the subject of another report in a companion paper.

The participants were stratified into three groups of intensity training based on their baseline's FDI MEP amplitude, adapted from Milot et al. (21): (1) low-intensity (LI: MEPs <50 μV); (2) moderate-intensity (MI: MEPs 50–120 uV) and (3) high-intensity (HI: MEPs >120 uV). Within each group, participants were then randomized to receive either real or sham tDCS.

The strength training program targeted the affected shoulder and elbow flexors, wrist extensors and grip muscles and consisted of lifting dead weights with the affected UL. The characteristics of the strength training program followed the recommendation of the American Stroke Association (ASA) of exercise prescription after stroke (45) (see Appendix for more details). Briefly, at the beginning of every week of training, each participant's 1RM (maximal load that could be lifted once), estimated by the 10 RM (46), was determined to allow a gradation of the training intensity. Depending on each participant's MEP amplitude, training started at 35, 50, or 70% of 1 RM for the low-, moderate- and high-intensity groups, respectively. Training intensity was increased by 5% each week to reach, at the end of the week 4, 50, 65, and 85%, for the low-, moderate- and high-intensity groups, respectively. Moreover, to ensure a similar training intensity within and between each MEP group, participants rated their perceived level of effort after each session of exercises on the Borg's Rating of Perceived Exertion (47). This self-perceived physical exertion scale is considered a valid tool to control for the intensity of the exercise after a stroke (17). Thus, for week #1, a score of 12–13/20 had to be reached whereas, for week #2–4, participants trained at a score of 15–16/20. The same training characteristics were applied for the grip muscles, but participants trained with a Jamar^®^ hydraulic hand dynamometer. The order in which each muscle group was trained was randomized each week. For the first 20 min of each training session, tDCS (Ottawa: HDCStim, Newronika, Milano, Italy; Montreal: NeuroConn, Llmenau, Germany; Sherbrooke: Soterix Medical, New York, USA) was applied using an anodal montage (12 sessions, 20 min, 2 mA). At each site, saline-soaked 5 × 7cm electrodes were used, with the active anode electrode placed over the lesioned primary motor cortex (M1) and the cathode electrode placed over the contralateral supra-orbital region. The determination of the M1 site was based on the localization of the FDI motor hotspot on the scalp (48). Sham tDCS was applied for the first 30 s to induce the same sensations as real tDCS (49), ensuring that the participants were blinded to the type of stimulation.

Prior to taking part in this study, all participants signed a consent form approved by the Research Ethics Committee (REC) of the CIUSSS de l'Estrie-CHUS (MP-22-2016-630) and Bruyère Research Ethics Committee (Protocol #M16-16-028).

### Statistical analysis

Descriptive statistics were used to characterize the sample. Sociodemographic characteristics of the training groups were compared using independent *t*-tests or Chi-squared tests, depending on the nature of the variables. Given the presence of multiple outcomes, the main dependent variables (i.e., FMA, BBT, Grip) were entered into a multivariate analysis of variance (MANOVA) to determine the effect of the intervention with Time (Pre vs. Post) as the repeated factor and tDCS (Real vs. Sham) and Training Group (LI, MI, HI) as the between-subject factors. Upon detection of main effects or interactions in the multivariate analysis, univariate tests were examined to determine the effect of each main dependent variable. Repeated measures ANOVA was performed for secondary outcome measures with tDCS (Real vs. Sham) and Training Group (LI, MI, HI) as the between-subject factors. If an interaction was noted, *post-hoc* analysis with a Bonferroni correction was applied to locate the difference. The significance level was set at *p* < 0.05 for all tests, and statistics were computed using IBM^®^ SPSS Statistics 25.

## Results

### Participants' characteristics

**Figure 1:**
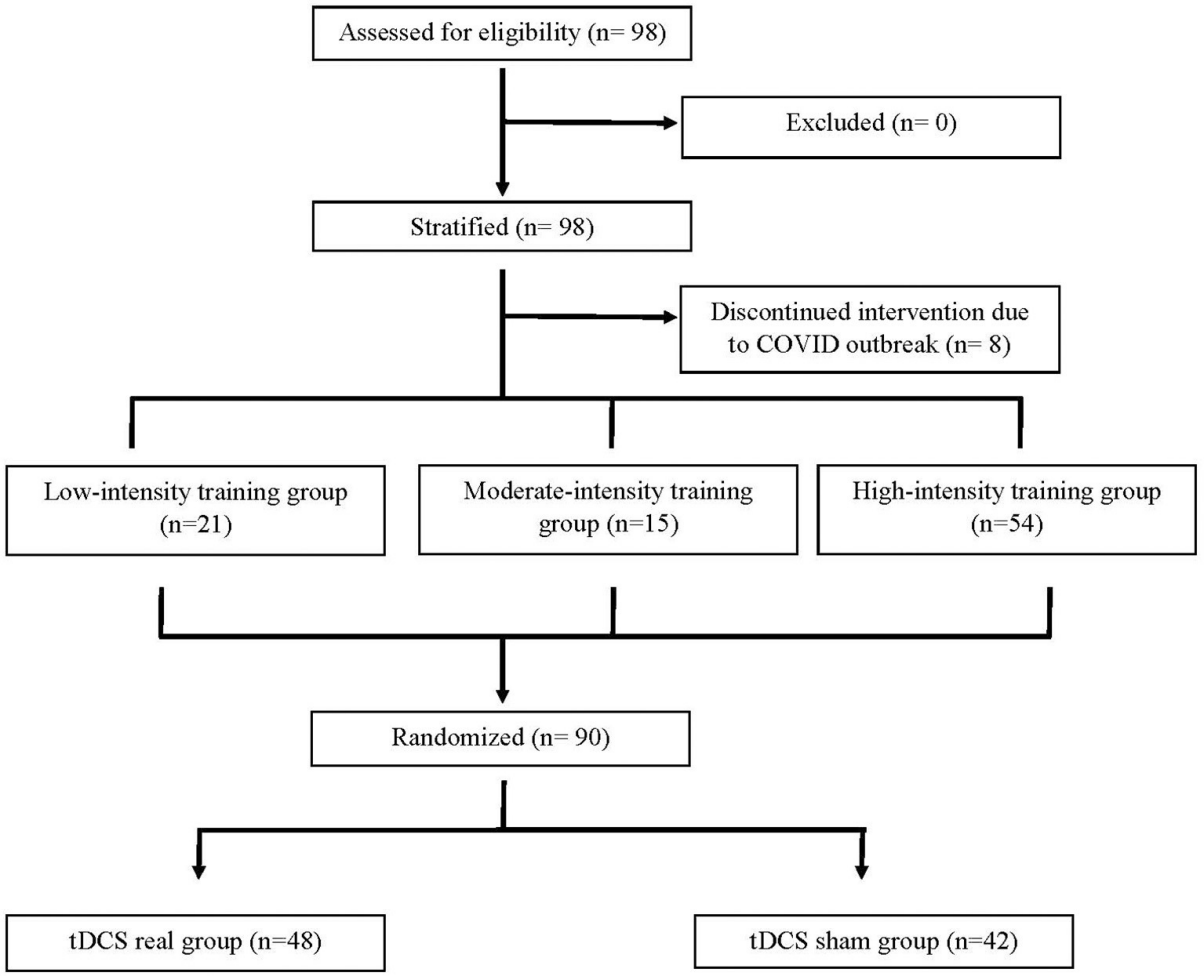
Study flow diagram.

**Figure 2:**
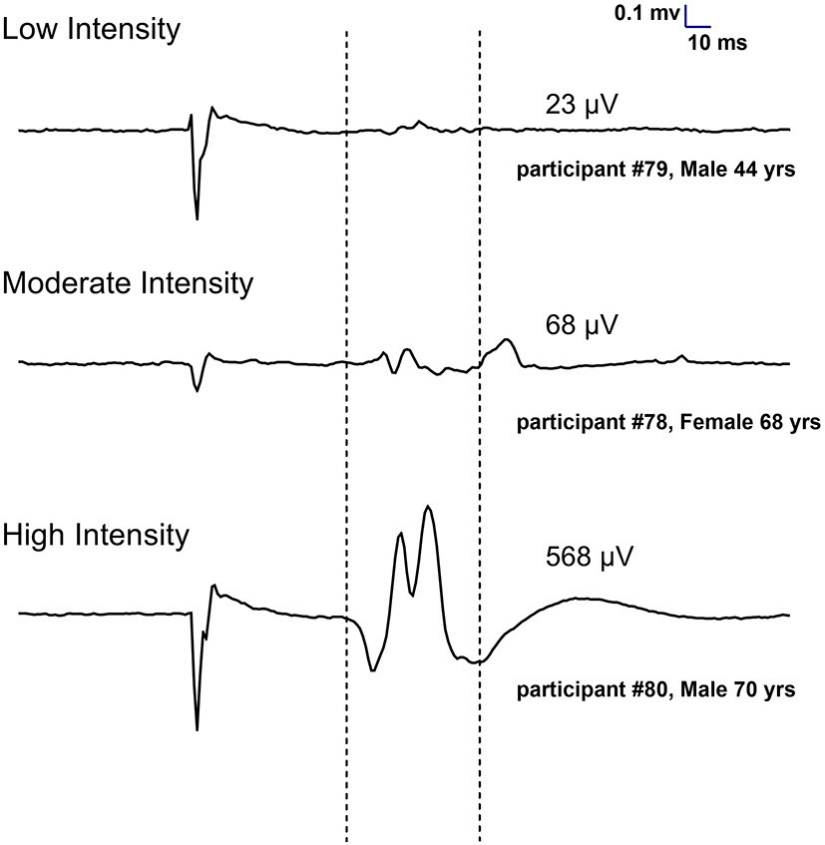
Individual examples of MEPs recorded at 130% of resting motor threshold in the first dorsal interosseous of the affected hand to assign participants to training groups (Low, Moderate and High intensity). Each trace represents an average of 10 trials.

As shown in Figure 1, 98 participants were initially screened for eligibility and stratified into the three training groups. However, because of the COVID outbreak, eight participants voluntarily withdrew before completing the intervention, leaving a total of 90 participants having completed the study. Based on MEP size in the affected arm, 21 participants were allocated to the LI group (mean MEP: 53 μV), 15 in the MI group (Mean MEP: 158 μV), and 54 in the HI group (Mean MEP: 717 μV) (see Figure 2). Note that for the LI group, no MEP amplitude was detected in the FDI and ECR for 6 participants. The randomization for tDCS allocated 48 participants to receive real stimulation, while 42 received the sham one. The sociodemographic characteristics of participants in each training group are presented in Table 1. All three groups showed comparable characteristics with respect to age (*p* = 0.37), time since stroke (*p* = 0.22) and male/female ratio (*p* = 0.62).

**Table 1:** Participants sociodemographic characteristics [mean (SD)] in each training group.

	**Training groups**		
	**LI** **(*n* = 21)**	**MI** **(*n* = 15)**	**HI** **(*n* = 54)**
Age (years)	63 (12)	68 (13)	65 (11)
Handedness (right/left)	19/2	13/2	49/5
Male/female	13/8	7/8	32/22
Time since stroke (years)	6 (7)	5 (3)	5 (4)
Type of stroke (ischemic/hemorrhagic/other)	21/0/0	13/2/0	42/9/3
Side of stroke (right/left)	9/12	6/9	33/21
Dominant/non-dominant affected side	12/9	9/6	19/35

LI, Low-intensity training group; MI, Moderate-intensity training group; HI, High-intensity training group.

### Effect of the tailored training intervention and tDCS on primary outcome measures

**Figure 3:**
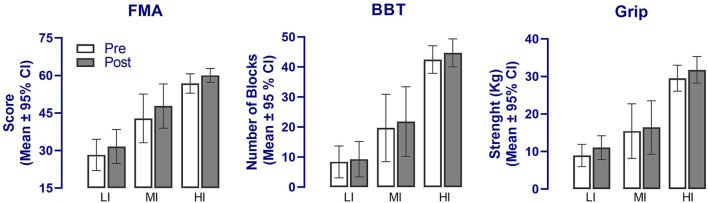
Changes in the main outcome measures following the upper limb tailored strength training and anodal tDCS intervention in the three training groups. A significant effect of “Time” was detected in all three primary outcomes (*p* < 0.01) but no interaction with groups. FMA, Fugl-Meyer Assessment; BBT, Box and Block test; LI, Low-intensity training group; MI, Moderate-intensity training group; HI, High-intensity training group.

Regarding primary outcomes, most participants, irrespective of their training groups, showed improvements after the 4-week intervention in terms of reduced impairments, manual dexterity and grip strength. These improvements can be appreciated by inspecting Figure 3, showing the means computed pre- and post-intervention for each outcome within each group. Each group experienced, on average, similar gains post-intervention both in terms of reduced impairment (FMA) and improved function (BBT and Grip strength). The multivariate analysis confirmed the large main effect of “Time” on primary outcomes [*F*_(3, 82)_ = 16.9, *p* < 0.001], but no “Training Group X Time” (*p* = 0.55) or “tDCS X Time” (*p* = 0.86) interactions. This analysis indicates that “Time” was the most critical factor influencing primary outcomes, while the lack of interactions confirmed that the different groups improved on the same level and that real or sham tDCS had no significant impact. The univariate analysis confirmed the significant effect of “Time” on each primary outcome measures [FMA, *F*_(1, 84)_ = 25.0, *p* < 0.001; $\eta_{\text{p}}^{2}$: LI, 0.3; MI, 0.5; HI, 0.2; BBT, *F*_(1, 84)_ = 9.8 *p* = 0.002; $\eta_{\text{p}}^{2}$: LI, 0.1; MI, 0.8; HI, 0.4; Grip strength, *F*_(1, 84)_ = 24.1, *p* < 0.001; $\eta_{\text{p}}^{2}$: LI, 0.5; MI, 0.3; HI, 0.3] as well as the lack of interaction with “Training Group” (*p* > 0.35) and “tDCS” (*p* > 0.54).

### Effect of the tailored training intervention and tDCS on secondary outcome measures

**Figure 4:**
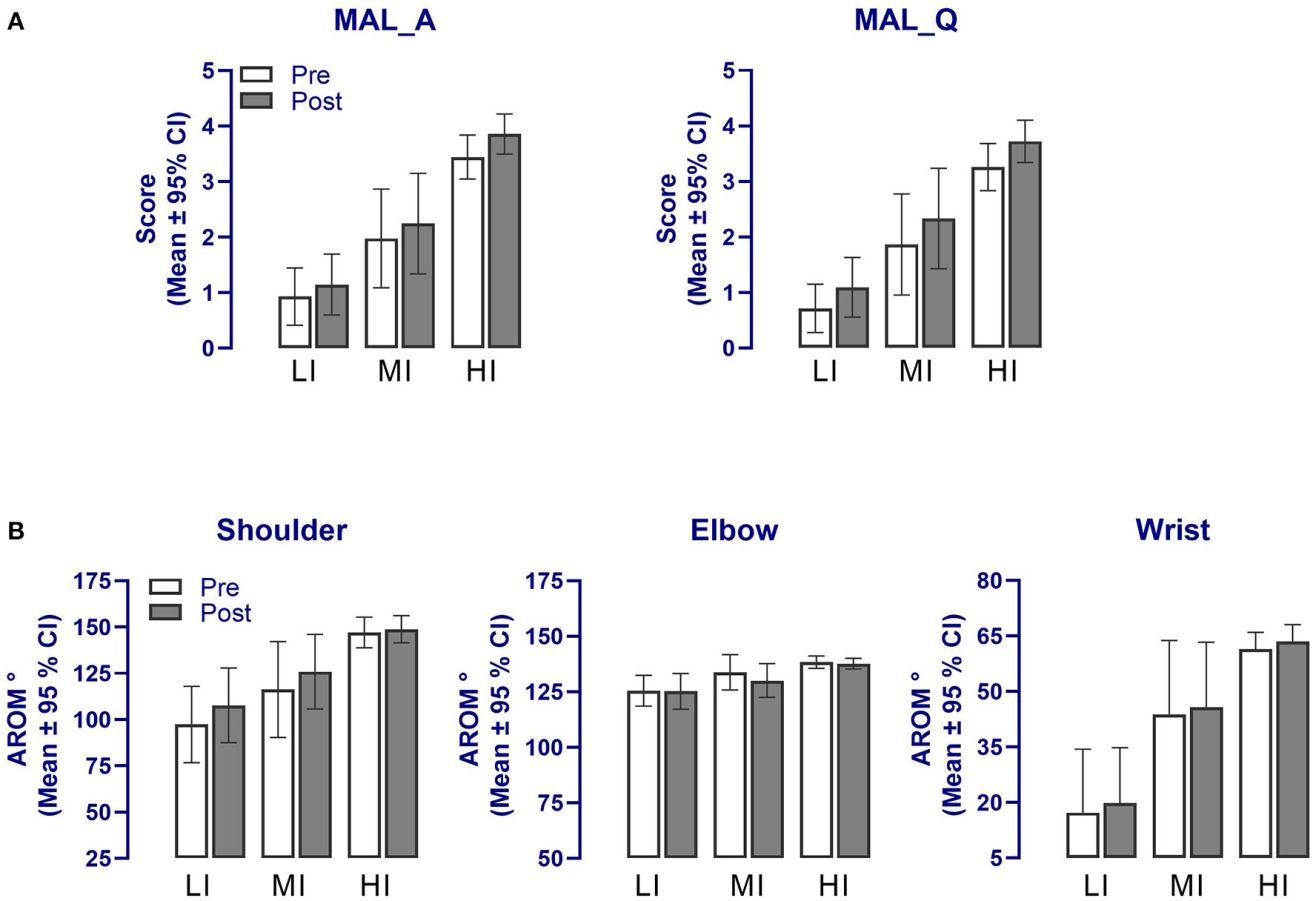
Changes in the secondary outcome measures following the upper limb tailored strength training and anodal tDCS intervention in the three training groups for **(A)** the MAL and **(B)** affected upper limb AROM. A significant effect of “Time” was detected in all secondary outcomes (*p* < 0.01) but no interaction with groups. Note that error bars for the wrist AROM are presented in one direction for clarity. MAL_A, Motor Activity Log amount of use; MAL_Q, Motor Activity Log quality of use; AROM, Active range of motion; LI, Low-intensity training group; MI, Moderate-intensity training group; HI, High-intensity training group.

Figure 4 compares each group's means computed pre- and post-intervention for the secondary outcomes. As for primary measures, the MANOVA revealed a significant effect of “Time” [*F*_(1, 84)_ = 11.5, *p* < 0.001] but no interaction with “Training Group” (*p* = 0.08) and “tDCS” (*p* = 0.58). As evident in Figures 4A,B, the effect of “Time” was largely attributable to changes in the MAL scores and the shoulder AROM, which was confirmed by the univariate analysis [MAL amount: *F*_(1, 84)_ = 24.5, *p* < 0.001; $\eta_{\text{p}}^{2}$: LI, 0.2; MI, 0.4; HI, 0.4; MAL quality: *F*_(1, 84)_ = 44.5, *p* < 0.001; $\eta_{\text{p}}^{2}$: LI, 0.4; MI, 0.6; HI, 0.5; AROMs: *F*_(1, 84)_ = 21.8, *p* < 0.001; $\eta_{\text{p}}^{2}$: LI, 0.3; MI, 0.3, HI, 0.03]. Further, for the shoulder AROM, a significant “Training Group X Time” interaction was noted (*F* = 4.6, *p* = 0.013), which reflected the fact that gains in AROM were noticeable only for the LI (*p* = 0.01) and MI (*p* = 0.04) groups (Bonferroni post-tests; see Figure 4B). Changes measured in the wrist and elbow AROM pre- and post-intervention were not significant (*F* < 3.2, *p* > 0.08, Figure 4B).

## Discussion

This study demonstrates improvements in arm motor function and performance in chronic stroke survivors who underwent a 4-week strength training intervention whose intensity was tailored based on the amplitude of MEPs in the affected limb. Our results also revealed that combining anodal tDCS with tailored strength training had no significant influence on outcome measures. Altogether, these results suggest that adjusting training intensity based on a neurophysiological marker of corticospinal tract integrity is critically important for optimizing the outcomes of strengthening exercises aiming to improve arm function post-stroke.

### Effects of the tailored strength training program on UL function and performance

As mentioned earlier, strengthening exercises are recommended post-stroke to improve residual weakness and restore function in the affected extremities (9). The present study provides further evidence of the benefits of strengthening programs for patients and highlight the importance of tailoring such interventions to optimize rehabilitation outcomes. Our tailored strength training intervention hardly compares with other training programs reported in previous studies (2, 11) since we are the first to have stratified participants in groups based on the size of MEP elicited in the affected arm. Thus, our study is the first to demonstrate that adjusting training intensity, using an index of corticospinal integrity based on MEP, is of critical importance to allow participants to experience gains in UL function, irrespective of their initial status in terms of severity. Indeed, our data showed that the vast majority of our participants experience improvements after the intervention (i.e., 70/90), as reflected in the FMA scores. Those who did not experience improvement (*n* = 20) consisted mostly of participant in the HI training group (17/20), whose FMA scores were already close to the maximum at baseline. Excluding those high performing individuals, 89% of our participants saw their FMA scores improved after the intervention. Also, in each training group, many participants experienced gains larger than the 5-point minimal clinically important difference (MCID) of FMA (50) (LI, 29%; MI, 47%; HI, 22%). For the BBT 60% of participants showed improved performance post-training, while for Grip strength, 77% improved post-training with 14% of participants exceeding the 5 kg MCID, as found in subacute stroke survivors (LI, 14%; MI, 7%; HI, 17%) (51). The improvements noticed in primary outcomes were also reflected in the secondary outcomes. For instance, both the MAL quantity and quality of UL use were improved post-intervention, indicating that participants reported increased use of their affected arm in real-life situations. In this respect, a substantial proportion of participants in each training group experienced a change greater than the 0.5-point clinically meaningful change in the MAL score post-stroke (52) (LI, 38%; MI, 26%; HI, 41%). In addition to the MAL, improvements were also detected in the AROM measured in the affected arm. These improvements were found for the shoulder only and were largely restricted to participants in the LI and MI groups. For the HI group, the lack of improvement likely reflected the fact that limitations in AROM were already minimal at baseline, leaving no room for further improvements. Given the crucial role played by the shoulder in the performance of daily tasks (53, 54), and considering the results of previous studies (11, 55), it is easy to see the link between the increased arm use reported by participants and the gains in shoulder AROM, both findings pointing to an improved arm function and usage post-intervention. Thus, both our primary and secondary measures converge to show that participants, irrespective of their training group allocation, experienced significant gains in their affected UL post-intervention that translated into improved function in daily life activities. These positive results stress the importance of considering MEP amplitude in post-stroke exercise prescription (20) to ensure that each individual is training at an optimal intensity, based on his specific recovery potential, knowing that intensity is a critical factor influencing motor improvement (17, 56).

### Lack of tDCS effects

Contrary to our prediction, the addition of anodal tDCS to our strength training program did not translate into greater gains for participants. tDCS is used to alter the brain excitability *via* modulation of cell membrane excitability and LTP-like plasticity mechanisms (57). Although it is unlikely that the response to tDCS post-stroke remains consistent from early to late recovery, here, tDCS was used in patients in the chronic stage to enhance neural excitability of the affected motor area and to promote neural plasticity (57). However, as mentioned, in this study, real or sham anodal tDCS of the affected hemisphere had no effect on both primary and secondary outcomes. While our tDCS protocol in terms of intensity and duration was in line with recent successful trials on tDCS in post-stroke populations (26), it is still possible that our chosen parameters were not optimal for every participant. Yet, in line with recent recommendation for tDCS in chronic stroke, our protocol targeted mostly moderately and mildly impaired individuals (26), who are considered good responders to tDCS, and was applied during and not after exercises (26). The lack of benefit of our tDCS protocol contrasts with positive results reported previously (28, 29, 33, 58). One possible cause to explain the negative finding may be in the intensity used. In our protocol we used 2 mA, but recent studies show that intensities up to 4 mA could yield to better efficacy in terms of current penetration and effects (26, 59), as observed in animal models (60). However, higher tDCS intensities may not always imply enhanced efficacy (61), and using high tDCS intensities raises concerns about safety and side effects (62). Also, a higher tDCS intensity has been shown to reverse tDCS-induced neuroplastic effects in healthy people (63–65). For example, Hassanzahraee et al. found that stimulation >1 mA for 26 min resulted in a reversal of anodal tDCS effects and was associated with a decrease in MEP amplitude and an increase in short-interval intracortical inhibition (63). However, these effects were also shown subsequently to be linked with the duration of the application (64), duration >26 min leading to a reversal of anodal stimulation. While it is still possible that the 2 mA intensity we used in our protocol might have led to a reversal, this possibility is less likely given that our duration was limited to 20 min. Beyond intensity and duration, one alternative cause for the lack of effects could come from the utilization of a unihemispheric instead of a bihemispheric montage. Indeed, there is evidence from systematic reviews that bihemispheric montage, with the anode place on the affected hemisphere, could provide an advantage over unihemispheric montage to promote motor learning after a stroke (66).

While there are many reports supporting tDCS effects post-stroke, our report is not the only one finding negative results and no benefit from the application of tDCS at the chronic stage of a stroke (15, 32, 67, 68). For example, Pavlova et al. reported no add-on effects of tDCS combined to a 4-week grip force task in their group of chronic stroke survivors (*n* = 11) (32). In a recent study by Hordacre et al., the authors propose a patient-tailored approach to deliver tDCS optimally after stroke (69). This new theoretical approach considers key characteristics to optimize tDCS response such as lesion site and extend as well as ipsilesional corticospinal tract (CST) integrity, the latest being the most important predictor to be considered within their algorithm. Although we measured ipsilesional CST integrity by means of baseline MEP amplitude, we did not find “tDCS Group X Time” interaction, indicating that tDCS had no influence regardless of the participants' status with regards to corticospinal integrity. This highlights the fact that there is currently insufficient evidence for the optimal effectiveness of tDCS due to stroke heterogeneity and unknown determinants affecting the tDCS outcomes. The fact that the stimulation of a neuronal (post-synaptic) population should be precisely timed with neuronal activity is also an important factor to consider in order to improve the efficacy of tDCS as to drive more efficiently neuronal reorganization (70). This is an important topic that deserves further investigation. Finally, since we did not find an add-on effect of anodal tDCS on UL training gains, it can be thought that tailoring training on each individual MEP amplitude is a very effective approach in inducing substantial gains in UL function after a stroke, as opposed to traditional rehabilitation exercises, resulting in the tDCS having no added value for this type of exercise.

Overall, the lack of benefit of tDCS in stroke rehabilitation demonstrates that optimal tDCS stimulation characteristics to boost recovery post-stroke are yet to be determined and that the design and adoption of more robust protocols across studies are needed (26, 69).

## Limitations

As the project was conducted throughout three different sites in Canada, a limitation of the study could be attributed to a possible lack of consistency in the data collection. To minimize this limitation, the research personnel involved at three sites were all trained before data collection. However, not all sites benefited from the same material (e.g., tDCS machine). Additionally, FMA scores were high in many participants pre-training, meaning that subtle improvements in motor impairment could not be quantified using this scale. However, the FMA is the recommended outcome to assess the various levels of impairment in chronic stroke survivors (71), and when combined with other clinical scales, such as the MAL, it can provide a more complete assessment of rehabilitation interventions destined to reduce impairment and activity limitations (72). Finally, a selection bias is present in our study, as individuals with major impairments post-stroke, who could not perform the training program, were excluded. This, in addition to the multiple exclusion criteria, may limit the generalizability of the results in the overall chronic stroke population.

## Conclusion

Tailoring exercise based on the participants' MEP amplitude, as assessed with TMS, translated into a marked improvement in motor function and performance of the affected UL. The current results add to the growing body of evidence stating that recovery can take place even at the chronic stage of a stroke, past the critical window for recovery considered to be traditionally within the first 3–6 months post-stroke and even in individuals having less recovered from their stroke, as in our low-intensity training group. However, the use of tDCS combined with exercises did not enhance treatment gains. The results of this study advance our knowledge on the usefulness of MEP stratification in stroke rehabilitation to prescribe tailored exercises at an appropriate level of intensity. More studies are needed to clarify the role of tDCS to enhance strength training interventions in chronic stroke survivors.

## Data availability statement

The raw data supporting the conclusions of this article will be made available by the authors, without undue reservation.

## Ethics statement

The studies involving human participants were reviewed and approved by Research Ethics Committee (REC) of the CIUSSS de l'Estrie-CHUS (MP-22-2016-630) and Bruyère Research Ethics Committee (Protocol #M16-16-028). The patients/participants provided their written informed consent to participate in this study.

## Author contributions

SP contributed to data collection, data analysis, and writing of the manuscript. YA and AR contributed to data collection and analysis. HC contributed to the protocol design and revision of the manuscript. FT, MHB, and MHM contributed to the protocol design, data analysis, and writing and revision of the manuscript. All authors contributed to the article and approved the submitted version.

## Funding

The project was funded by the Brain Canada Foundation along with Fonds de recherche du Québec-Santé, la Fondation Vitae, Centre de recherche interdisciplinaire en réadaptation du Montréal métropolitain (CRIR), and Jewish Rehabilitation Hospital Foundation. This work was supported by funds from the Canadian Foundation for Innovation grant number 34277 [MHB]. The research was undertaken thanks in part to funding awarded to SP from the Canada First Research Excellence Fund, awarded to McGill University as part of the Healthy Brains for Healthy Lives (HBHL) initiative and two CRIR bursary Master Fellowships.

## Conflict of interest

The authors declare that the research was conducted in the absence of any commercial or financial relationships that could be construed as a potential conflict of interest.

## Publisher's note

All claims expressed in this article are solely those of the authors and do not necessarily represent those of their affiliated organizations, or those of the publisher, the editors and the reviewers. Any product that may be evaluated in this article, or claim that may be made by its manufacturer, is not guaranteed or endorsed by the publisher.
