# Supplementary material for: Effect of a tailored upper extremity strength training intervention combined with direct current stimulation in chronic stroke survivors: A Randomized Controlled Trial
Source: Front Rehabil Sci. 2022 Aug 3;3:978257. doi: 10.3389/fresc.2022.978257 (PMC9397935; doi:10.3389/fresc.2022.978257)
Supplement: Supplementary file 1 [file Data_Sheet_1.docx]

**Strength training program characteristics**

**General characteristics**

1. Number of repetitions : 10
2. Series : 3
3. Rest between series: 2 minutes
4. Warm up: 5 minutes
5. Cool-down: 5 minutes
6. Duration of training : 60 minutes
7. Frequency : 3 times/week
8. Total duration: 4 weeks

**Positioning of trained muscle groups**

1. **Wrist extensors**

Starting position

- Subject is sitting
- Affected forearm in pronation and stabilized on a table or an armrest
- Affected hand is free at the end of the table or armrest

Instruction to participants

“Move your hand up slowly and as high as possible. Then move your hand down slowly while controlling the movement of your hand.”

1. **Elbow flexors**

Starting position

- Subject is sitting
- Affected elbow at 90^0^ and forearm in supination

Instruction to participants

“Bend your elbow and bring your forearm as close as possible to your shoulder. Then, slowly straighten your elbow while controlling the movement of your forearm until your forearm goes back to its starting position.”

1. **Shoulder flexors**

Starting position

- Subject is sitting
- Affected elbow in extension and forearm in neutral position
- Affected shoulder slightly flexed and in 0°of abduction

Instruction to participants

“By keeping your elbow as straight as possible, lift your arm as high as possible. Be sure to keep your arm in line with your body and do not move your back from front to back or sideways during the movement. Then, bring back your arm slowly to its starting position by controlling the movement of your arm.”

1. **Grip muscles**

Starting position

- Subject is sitting
- Affected elbow at 90^0^, forearm in neutral position, affected shoulder slightly flexed and in 0° of abduction
- Handle of the dynamometer positioned so that the distal phalanges embody the handle

Instruction to participants

“Squeeze the handle by closing your hand around it and maintain your squeeze for 3 seconds. Then release.”
